# The Role of Glutathione Peroxidase 4 in Atherosclerosis: Role and Therapeutic Potential

**DOI:** 10.31083/RCM47324

**Published:** 2026-04-17

**Authors:** Zixin Guo, Xiaoyu Wei, Tianyi Gu, Hangyan Guo, Shengyu Hua

**Affiliations:** ^1^Graduate School of Tianjin University of Traditional Chinese Medicine, 301617 Tianjin, China; ^2^College of Chinese Medicine, Tianjin University of Traditional Chinese Medicine, 301617 Tianjin, China

**Keywords:** GPX4, ferroptosis, atherosclerosis, lipid peroxidation, endothelial dysfunction

## Abstract

Atherosclerosis, characterized by abnormal lipid metabolism and inflammation, constitutes the fundamental pathological basis for the development of cardiovascular lesions. Ferroptosis, a recently discovered novel form of cell death, is linked to disturbances in iron metabolism and lipid peroxidation; meanwhile, an association with various cardiovascular diseases, including heart failure, myocardial infarction, and atherosclerosis, has also been confirmed. Glutathione peroxidase 4 (GPX4) is an important component of the antioxidant system that plays a key role in maintaining iron homeostasis and inhibiting ferroptosis. Ferroptosis triggered by GPX4 inactivation can also further activate pyroptosis pathways by releasing proinflammatory signals, thereby collectively exacerbating inflammation and the progression of atherosclerotic plaques. Therefore, further investigations into the function of GPX4 in atherosclerosis may facilitate the development of novel diagnostic and therapeutic approaches, as well as drug development targets for the prevention and prognosis of related cardiovascular diseases. Moreover, the activation of GPX4 or the supplementation with its coenzyme, glutathione (GSH), may emerge as a promising new therapeutic strategy. This review summarizes the structure and function of GPX4 and the role of this enzyme in iron toxicity and atherosclerosis.

## 1. Introduction

Atherosclerosis (AS), a chronic vascular disease marked by inflammation, disrupted lipid metabolism, and endothelial dysfunction, continues to be a major global cause of death, even though its incidence has declined in some regions. This persistent clinical impact stems from its insidious progression and severe complications, such as myocardial infarction and stroke [1, 2, 3]. As AS disproportionately affects aging populations, ongoing research into its complex pathophysiology is crucial for developing more effective treatments.

Ferroptosis is a unique form of cell death driven by iron accumulation and lipid peroxidation, distinguishing it from other cell death mechanisms like apoptosis, necroptosis, pyroptosis, and autophagy. Biochemically, ferroptosis occurs when intracellular glutathione levels drop and glutathione peroxidase 4 (GPX4) activity is impaired, resulting in an unchecked accumulation of lipid peroxides that GPX4 would typically reduce [4, 5]. The process is characterized by increased lipid reactive oxygen species (ROS) and peroxidation of polyunsaturated fatty acids (PUFAs) in the cell membrane, hallmarks of ferroptosis [6].

Recent research highlights ferroptosis as a critical factor in cardiovascular disease, particularly AS [7]. GPX4 plays a pivotal role in this context by maintaining redox balance through neutralizing toxic lipids, offering dual protection against both ferroptosis and atherosclerotic progression [8]. In this review, we examine GPX4’s structure and function, its role in ferroptosis and AS, explore the relevant signaling pathways, and discuss how targeting these pathways could lead to new therapeutic strategies for managing cardiovascular disease.

## 2. Ferroptosis and the Pathogenesis of AS

**Fig. 1.**
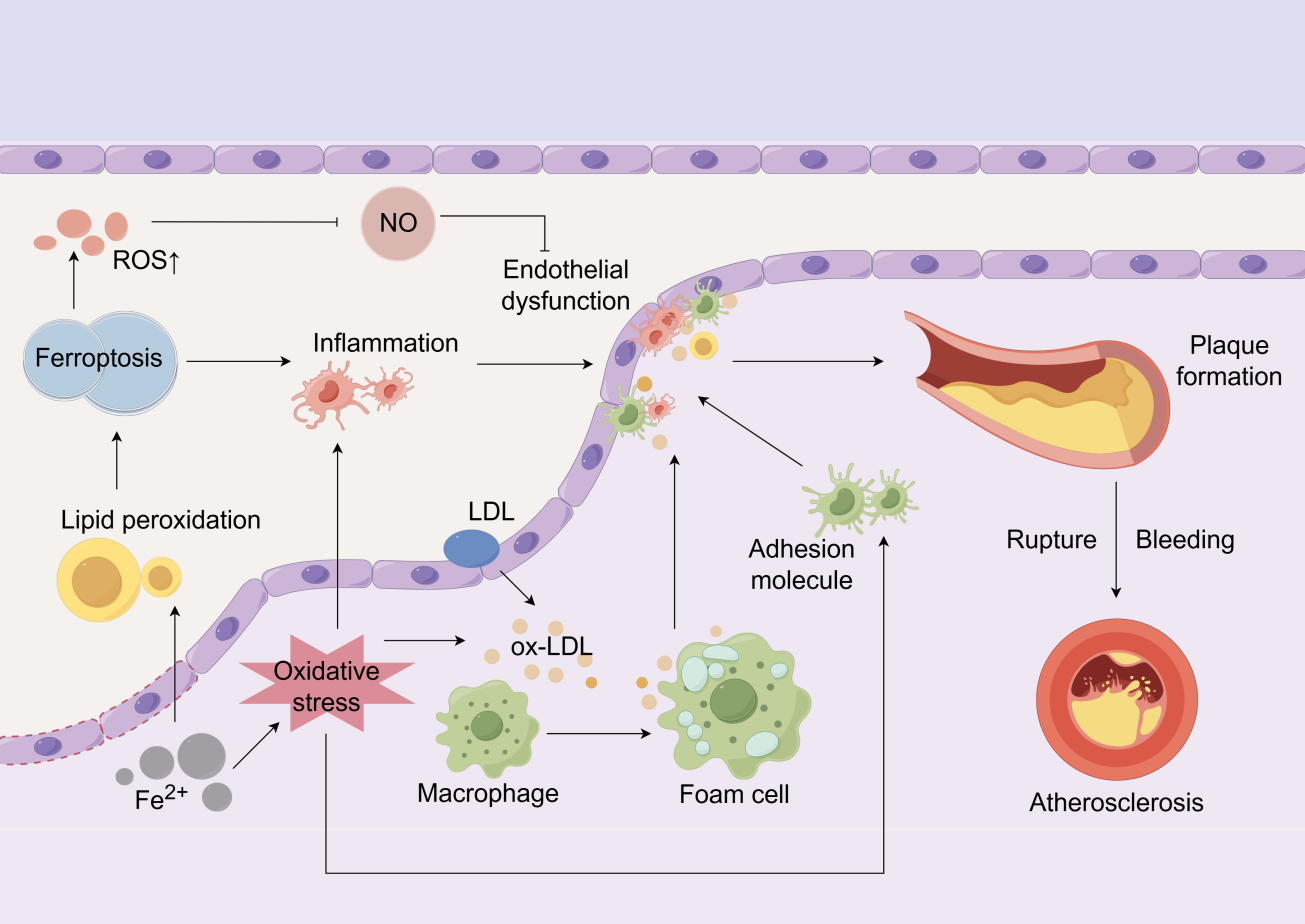
**Ferroptosis is associated with the pathogenesis of atherosclerosis**. Endothelial dysfunction lets LDL penetrate and be oxidized to ox-LDL; Fe^2+^ buildup heightens oxidative stress and lipid ROS, prompting macrophages to form foam cells that undergo ferroptosis, spill inflammatory mediators, recruit more immune cells and enlarge the necrotic core, together expanding the plaque until the fibrous cap ruptures, platelets adhere and bleeding/thrombosis ensue. Concurrently, excessive ROS caused by ferroptosis inhibits NO activity, further damaging the endothelium and exacerbating inflammation and endothelial dysfunction. LDL, low-density lipoprotein; ROS, reactive oxygen species; NO, nitric oxide. The Fig. 1 was created using the following online platform: https://www.figdraw.com. Copyright Code: AIPRAffdfa. Manufacturer Location: Hangzhou City, Zhejiang Province, China.

AS is a chronic inflammatory disease resulting from lipid metabolism dysfunction. It progresses through lipid accumulation, inflammatory cell infiltration, and plaque instability, ultimately leading to thrombosis [9]. Notably, the cell death contributing to the formation of this necrotic core in AS is now partly attributed to ferroptosis. The pathogenesis of AS involves the dysfunction and death of key vascular cells, including endothelial cells [10], vascular smooth muscle cells (VSMCs) [11], and macrophages [12]. Recent studies have revealed that several hallmarks of ferroptosis, such as significant iron accumulation [13], and GPX4 inhibition [14], are involved in AS pathogenesis. In AS, iron overload in macrophages and endothelial cells can promote oxidative stress and foam cell formation, while also increasing the release of inflammatory mediators, thereby aggravating plaque development—effects that can be mitigated by iron chelation [15]. Hence, ferroptosis interacts with these pathological mechanisms, collectively driving the onset and progression of AS (Fig. 1).

### 2.1 Ferroptosis and Lipid Peroxidation

The adverse effects of excess iron on vascular homeostasis are well-documented, linking iron overload, oxidative stress, and endothelial dysfunction to AS [16]. Ferroptosis, characterized by an oxidative-antioxidant imbalance [17], shares key features with advanced AS lesions, including lipid peroxidation and iron deposition [18]. There are two types of lipid peroxidation involved in ferroptosis: enzymatic and non-enzymatic. Non-enzymatic lipid peroxidation is mainly triggered by hydroxyl radicals generated from the interaction between transition metal ions like Fe^2+^ and ROS [19]. Within AS plaques, this reaction is fueled by the labile iron pool in macrophages and endothelial cells, amplifying local oxidative damage. Lipoxygenases (LOXs) are key enzymes in enzymatic lipid peroxidation reactions [20].

During peroxidation, membrane lipids rich in PUFAs are particularly susceptible to free radicals and LOXs. LOXs, which contain iron, facilitate the oxidation of PUFAs, leading to the production of hydroperoxide derivatives that ultimately disrupt membrane integrity [21, 22]. For instance, Yang *et al*. [23] demonstrated that *LOX* gene knockout confers protection against Erastin-induced ferroptosis, suggesting a role for LOXs in this process. Moreover, GPX4 inhibition results in unchecked PUFA oxidation and fatty acid radical generation, culminating in ferroptosis [23]. Treatment of *ALOX15* knockdown cells with ferroptosis inducers significantly reduced cell mortality, and ALOX15 can cooperate with GPX4 to regulate ferroptosis [24]. The lipid-rich environment of atherosclerotic plaques provides ample substrate for these LOX-mediated and non-enzymatic peroxidation pathways, directly linking ferroptosis to plaque vulnerability.

Dyslipidemia further connects ferroptosis to AS progression. In ApoE^-⁣/-^ mice, a high-fat diet (HFD) exacerbates both AS severity and ferroptosis level [25]. After administering the ferritin inhibitor Fer-1, Fe^2+^ and lipid ROS levels significantly decreased, cell viability was restored, lipid peroxidation was alleviated, and ferroptosis was inhibited. Additionally, inhibiting ferroptosis led to significant reductions in total cholesterol, low-density lipoprotein (LDL) cholesterol, and triglyceride levels in AS mice, consistent with the efficacy of clinical hyperlipidemic drugs [26].

### 2.2 Ferroptosis Induces Endothelial Dysfunction

AS is primarily triggered by the accumulation of specific plasma lipoproteins, such as low-density lipoproteins (LDLs) and remnants of triglyceride-rich lipoproteins, in the arterial intima [27]. This attracts monocytes that bind to activated endothelial adhesion molecules, enter the intima, and differentiate into macrophages, which then become lipid-laden foam cells. Activated endothelial cells and macrophages secrete chemokines and growth factors that recruit VSMCs to form a fibrous cap [28, 29]. The integrity of the endothelial layer is crucial, and its dysfunction is widely recognized as the initial step in AS development [30].

Ferroptosis directly drives this critical endothelial dysfunction. Endothelial cells regulate vascular tone and homeostasis by producing the vasodilator nitric oxide (NO). Redox imbalance is a major contributor to endothelial dysfunction; excess ROS reduces NO bioavailability and impairs endothelium-dependent vasodilation, leading to vascular aging and AS [31, 32]. By definition, ferroptosis propagates massive lipid ROS generation, directly contributing to this pathogenic environment. In ApoE⁻/⁻ mice, chronic iron overload exacerbates atherogenesis by inducing oxidative stress and endothelial dysfunction [33]. Thus, the ferroptosis-driven surge in oxidative stress not only alters vascular tone but also increases leukocyte adhesion and lipid peroxidation, creating a perfect storm for AS initiation and progression [34].

### 2.3 Ferroptosis Exacerbates the Inflammatory Response

AS is widely acknowledged as a chronic inflammatory disease, with substantial evidence highlighting inflammation’s pivotal role in its pathogenesis, where macrophage polarization significantly influences disease outcomes [35]. Active M1 inflammatory macrophages are key drivers of chronic inflammation in AS [36]. Ferroptosis acts as a powerful amplifier of this inflammatory process. Excess iron within macrophages boosts reactive ROS production and promotes pro-inflammatory M1 polarization, leading to increased cytokine secretion that intensifies the inflammatory response [37, 38]. Moreover, M1 macrophages suffer from mitochondrial dysfunction, which hinders their polarization into anti-inflammatory M2 macrophages [39], thereby perpetuating a pro-atherogenic state [40].

Pattern recognition receptors, such as toll-like receptors (TLRs) and nucleotide-binding oligomerization domain-like receptors (NLRs), initiate immune responses by recognizing damage-associated molecular patterns (DAMPs) released from damaged or dying cells [41]. Ferroptotic cells release a plethora of DAMPs and oxidized lipids that can activate these receptors. This activation influences macrophage polarization, resulting in the release of pro-inflammatory factors that undermine plaque stability [42, 43, 44]. This inflammatory process is not confined to macrophages. Recent studies reveal that phenotypic changes in VSMCs are also strongly linked to AS-associated inflammation [45]. For instance, TLR4 mediates the osteogenic phenotypic transformation of VSMCs induced by oxidized low-density lipoprotein (ox-LDL) and promotes vascular calcification through nuclear factor kappa-B (NF-κB) activation [46]. Dysregulation of fungal metabolites also disrupts intestinal barrier function, activates immunity, and results in metabolic dysfunction and vascular inflammation [47, 48].

During AS progression, a vicious cycle exists between lipid modification and inflammation, with ferroptosis at its core. Studies have shown that ferroptosis is inherently pro-inflammatory. Iron overload promotes the release of inflammatory mediators like interleukin (IL)-1β and IL-18, creating a pro-atherogenic microenvironment within the plaque [49]. The release of DAMPs from ferroptotic cells further activates inflammatory pathways in surrounding cells, establishing a self-perpetuating loop of inflammation and cell death that drives AS progression [50].

### 2.4 Ferroptosis Promotes Foam Cell Formation

Macrophages play dual roles in AS pathogenesis, with both beneficial and detrimental effects [51]. In advanced stages, excessive macrophage death and impaired phagocytosis trigger secondary necrosis, releasing inflammatory contents and promoting the formation of a lipid-rich necrotic core that destabilizes plaques [52, 53, 54]. The progression of AS lesions depends on the local proliferation of macrophages and their uptake of lipoproteins to form foam cells [55, 56].

Notably, elevated iron levels are significantly observed in both human and animal atherosclerotic lesions, with accumulation being most pronounced in macrophages and positively correlated with lesion severity [57, 58]. Hepcidin, a regulator of iron metabolism, maintains iron homeostasis by inhibiting the iron exporter ferroportin (Fpn) [59]. In LDLR^-⁣/-^ mice, hepcidin deficiency reduces iron content in aortic macrophages and the number of pro-inflammatory M1 macrophages [60]. Conversely, ferroportin1 deficiency accelerates AS progression in mice, associated with increased ROS, enhanced inflammatory responses, and greater plaque lipid content [61], underscoring the critical role of macrophage iron export in protecting against ferroptosis and AS.

*In vitro* studies directly link iron to foam cell pathophysiology. Yang *et al*. [62] used ox-LDL to induce ferroptosis in macrophages and observed that the ferroptosis inhibitor Fer-1 could reduce foam cell formation by regulating lipid levels in macrophages while enhancing the expression of glutathione (GSH) and GPX4. This demonstrates how iron overload can directly trigger ferroptosis in the key AS cells. Furthermore, Zhong *et al*. [63] found that aldehyde dehydrogenase 2 (ALDH2) interacts with LDL receptors to promote lipid deposition and foam cell formation. Given that LDL receptor-mediated uptake is a primary pathway for macrophages to absorb LDL and become foam cells, any process that increases this uptake or concurrently induces oxidative stress, like ferroptosis, synergistically promotes atherogenesis. Moreover, studies indicate that iron overload exacerbates AS by inciting inflammation and enhancing glycolysis within macrophages, further integrating ferroptosis with core metabolic pathways in plaque development [64].

## 3. GPX4 and Ferroptosis in Atherosclerosis

Ferroptosis is an iron-dependent, non-apoptotic form of cell death resulting from lipid peroxidation, with GPX4 being its main negative regulator [65]. This process essentially reflects a breakdown of intracellular redox balance, leading to the accumulation of PUFAs on cell membranes and subsequent oxidative damage [66]. In atherosclerotic plaques, this mechanism is thought to cause the death of key cells like macrophages and VSMCs, directly contributing to necrotic core formation and plaque destabilization. Morphologically, ferroptosis is marked by compromised plasma membrane integrity, swelling of the cytoplasm and organelles, and distinct mitochondrial changes, including shrinkage, loss of cristae, and outer membrane rupture [67, 68].

### 3.1 GPX4: Structure and Function

The GPx family consists of vital antioxidant enzymes found in all living organisms, playing crucial roles in maintaining oxidative balance [69]. As the principal selenoproteins in humans, GPxs incorporate the rare amino acid selenocysteine, which provides potent antioxidant activity essential for health and disease [70]. Among the eight GPx isoforms identified in mammals, four selenium-dependent isozymes (GPX1–4) have distinct tissue distributions, structural features, and substrate specificities. While GPX1–3 function as homotetramers, GPX4 operates as a monomer, a unique property that underlies its specialized functions [71, 72].

Originally named phospholipid hydroperoxide GPx, GPX4 uniquely reduces complex hydroperoxides, including lipid hydroperoxides and cholesterol derivatives, unlike other GPxs that primarily target small organic peroxides [73, 74]. This ability is particularly important in AS, where the oxidation of cholesterol and phospholipids in LDL is a key initiating event [75]. Thus, GPX4 is a critical defense against the lipid peroxides that drive both foam cell formation and ferroptosis in plaques. GPX4 has a typical thioredoxin structure and contains catalytically active tetramers, similar to other GPxs, which consist of selenocysteine, glutamine, tryptophan, and asparagine [76, 77]. Redox catalysis occurs on selenocysteine or cysteine residues [78]. The biological importance of GPX4 was highlighted when *GPX4* knockout was found to cause embryonic lethality [79], and subsequent studies demonstrated that GPX4 depletion induces the most severe cell death among GPx isoforms [80]. Mechanistically, GPX4 inactivation triggers ferroptosis through uncontrolled lipid peroxidation [81], confirming its central role in this cell death pathway. The embryonic lethality of *GPX4* knockout highlights its indispensable role in cellular survival, a function equally critical for maintaining the viability of vascular cells under the pro-oxidant stress of the atherosclerotic microenvironment.

### 3.2 GPX4 Represents a Primary Regulatory Site of Ferroptosis in AS Pathogenesis

Ferroptosis arises from a complex interplay between excessive ROS production, GSH depletion, and iron overload [82]. While mitochondrial respiration generates ROS as byproducts, these molecules can initiate ferroptosis by oxidizing membrane phospholipids. GPX4 acts as the key defense mechanism by using GSH to detoxify lipid peroxides, thereby preserving membrane integrity and cellular homeostasis [83]. In AS, ROS from dysfunctional mitochondria, GSH depletion due to metabolic stress, and iron overload, especially in macrophages, create an environment where GPX4 can become overwhelmed. Additionally, GPX4 inactivates lipid-metabolizing enzymes to reduce ROS production, exerting an anti-ferroptosis effect [84].

Countermeasures against ferroptosis include iron chelators, lipophilic antioxidants, lipid peroxidation inhibitors, and PUFA depletion [66]. Although the final step leading to ferroptosis remains unclear, the appearance of specific lipid peroxidation products precedes cell disintegration and death. Therefore, substances and conditions that inhibit lipid peroxidation are potential strategies to prevent ferroptosis and associated disease [85, 86]. For AS, this suggests that therapeutic strategies aimed at enhancing GPX4 function or mimicking its activity could directly target the core pathological process of cell death within plaques.

Experimental models consistently demonstrate that GPX4 deficiency induces ferroptosis across various cell types—mesenchymal cells [87], T cells [88], and neurons [89], all of which accumulate significant lipid peroxides upon GPX4 depletion. This is also true for the major cell types involved in AS. For example, inhibiting GPX4 expression has been shown to promote macrophage ferroptosis and exacerbate atherosclerotic plaque formation, directly linking this axis to the disease [90]. Emerging research reveals that multiple metabolic pathways converge on ferroptosis regulation, particularly through GPX4 modulation. Isoprenyl pyrophosphate, a product of the mevalonate pathway, effectively stabilizes selenocysteine-specific tRNA, promoting GPX4 synthesis and regulating ferroptosis. Furthermore, inhibiting the mevalonate pathway with statins has been shown to impair efficient GPX4 translation, leading to ferroptosis [91]. This presents an intriguing paradox in cardiovascular therapy: while statins are the cornerstone of AS treatment for their lipid-lowering effects, their potential to inhibit GPX4 translation suggests a complex, context-dependent relationship with ferroptosis that warrants further investigation in the vascular context.

### 3.3 GPX4-Related Signaling Pathways in Ferroptosis

**Fig. 2.**
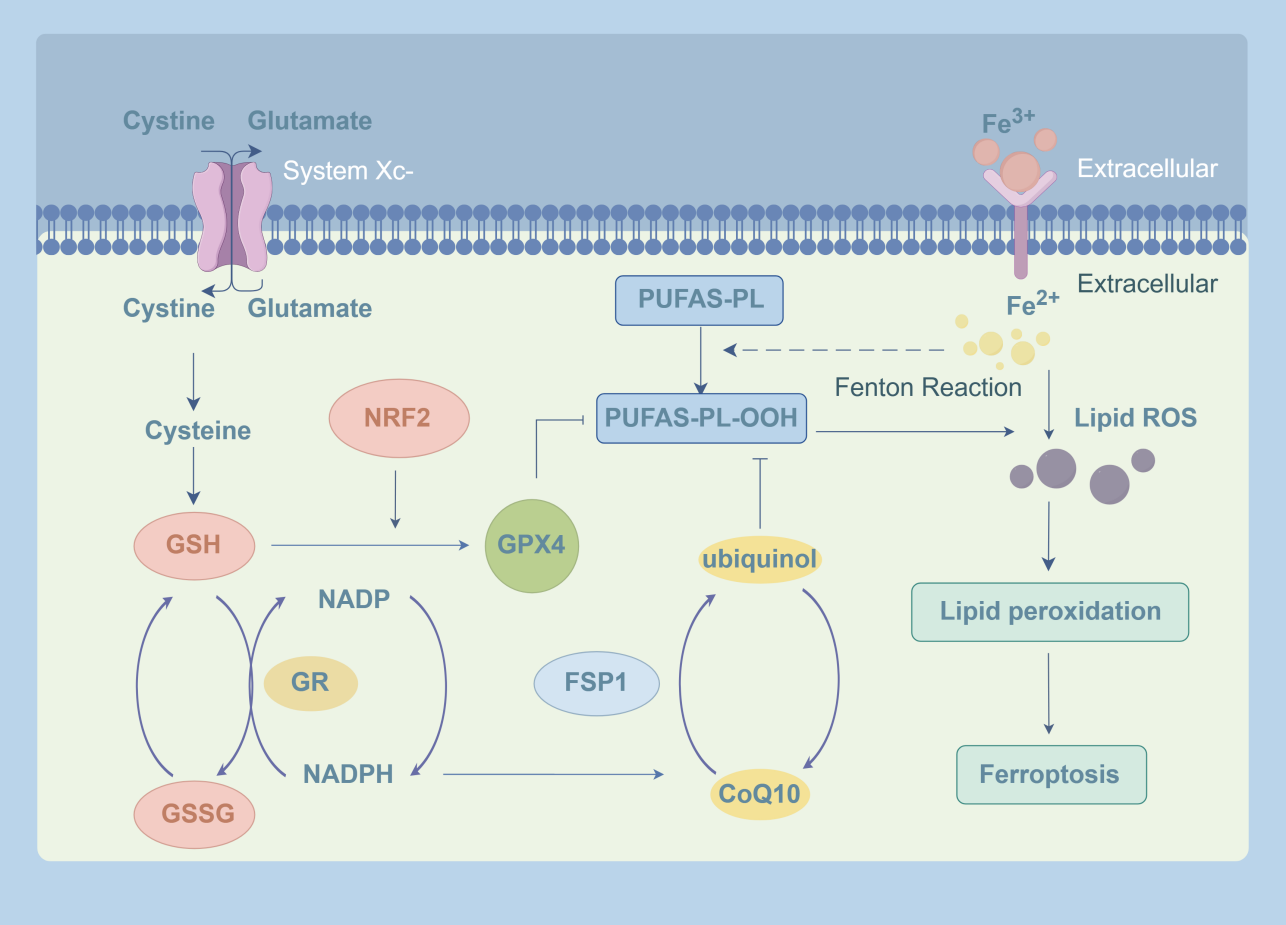
**GPX4-related signaling pathways in ferroptosis**. Intracellular Fe^2+^ and H_2_O_2_ attack polyunsaturated fatty acid-phospholipids (PUFA-PLs) via the Fenton reaction, generating phospholipid-polyunsaturated fatty acid-hydroperoxide (PUFA-PL-OOH), which is the primary source of lipid ROS and triggers ferroptosis. System Xc- transports cystine for GSH synthesis. GPX4 then utilizes GSH to reduce PUFA-PL-OOH into non-toxic products, forming GSSG. GSSG is subsequently regenerated back to GSH by GR, using NADPH as a substrate. Activation of Nrf2 enhances the expression of both GSH and GPX4, thereby boosting the antioxidant capacity. Independently, FSP1 reduces CoQ10 to ubiquinol using NADPH, which cooperates with GPX4 to suppress lipid peroxidation and protect cells from ferroptosis. GPX4, glutathione peroxidase 4; GSH, glutathione; GSSG, oxidized glutathione; GR, glutathione reductase; NADPH, nicotinamide adenine dinucleotide phosphate oxidase; Nrf2, nuclear factor erythroid 2; FSP1, ferroptosis suppressor protein 1; CoQ10, convert coenzyme Q10. The Fig. 2 was created using the following online platform: https://www.figdraw.com. Copyright Code: AUWSA04004. Manufacturer Location: Hangzhou City, Zhejiang Province, China.

Ferroptosis activation mainly involves three classical pathways: the System Xc-/GPX4 axis, lipid metabolism, and iron metabolism [92]. Moreover, the ferroptosis suppressor protein 1 (FSP1)-convert coenzyme Q10 (CoQ10)-nicotinamide adenine dinucleotide phosphate oxidase (NADPH) pathway and the kelch like ECH associated protein 1 (Keap1)-nuclear factor-erythroid 2 related factor 2 (Nrf2) pathway are also closely linked to GPX4 (Fig. 2). As all these pathways promote intracellular ROS production and lipid hydroperoxide (LPO) to induce ferroptosis, there may be potential synergies or correlations among them and their protein factors, enabling cooperative action against ferroptosis [93]. However, their relative significance and interactions in the specific context of AS are not fully understood. Clarifying this hierarchy is crucial for developing targeted therapies.

#### 3.3.1 System Xc-/GSH/GPX4 

The System Xc-/GSH/GPX4 axis is the canonical pathway for regulating ferroptosis [94]. The cystine/glutamate antiporter system (System Xc-), composed of solute carrier family 7 member 11 (SLC7A11) and solute carrier family 3 member 2 subunits, imports cystine for GSH synthesis, maintaining the redox capacity necessary for GPX4 activity [66]. The functionality of System Xc- heavily relies on SLC7A11 expression, as its downregulation impairs cystine import, depletes GSH stores, and ultimately inactivates GPX4 [95, 96].

In AS, the importance of this axis is well-supported by evidence. Activating the Nrf2/SLC7A11/GPX4 pathway inhibits ox-LDL-induced ferroptosis in vascular smooth muscle cells (VSMCCs) and reduces foam cell formation [97]. Macrophages, which are critical for plaque progression and have a high cysteine demand, may be highly sensitive to System Xc- inhibition. Cysteine deficiency enhances pro-inflammatory gene expression in macrophages, whereas increased cysteine uptake mediated by SLC7A11 generates superoxides to suppress excessive inflammation [98]. Notably, GPX4 can independently suppress ferroptosis by directly reducing lipid peroxides, even under GSH-depleted conditions [58]. This suggests that directly activating GPX4 may be a more effective strategy than targeting the upstream System Xc- in the GSH-depleted environment of advanced plaques.

#### 3.3.2 FSP1-CoQ10-NADPH Pathway

The FSP1-CoQ10-NADPH pathway functions as a GPX4-independent system for suppressing ferroptosis [99]. FSP1, initially identified as a pro-apoptotic factor, protects against ferroptosis induced by GPX4 deficiency [100]. Myristoylated FSP1 is recruited to the plasma membrane, where it uses NADPH to CoQ10 into ubiquinol, a potent lipophilic antioxidant that terminates lipid peroxidation chains [101, 102].

The significance of this pathway in AS is an emerging and vital area of research. Pharmacological studies show that idebenone (a CoQ10 analog) can reduce cardiotoxicity by stabilizing FSP1 and preventing ferroptosis. This indicates that enhancing the FSP1-CoQ10 axis could be a viable therapeutic approach, especially in cells with compromised GPX4 function. The finding that combined inhibition of FSP1 and GPX4 synergistically induces ferroptosis in cancer cells [103] suggests that dual-target therapies could be highly effective in AS, but their potential toxicity must be carefully evaluated. CoQ10 deficiency, observed in models of mitochondrial dysfunction [104, 105], may increase cellular vulnerability to ferroptosis. Given the documented mitochondrial dysfunction in AS, the FSP1-CoQ10 axis may serve as a critical compensatory mechanism, and its failure could accelerate disease progression.

#### 3.3.3 Keap1-Nrf2 Pathway

Nuclear factor erythroid 2 (Nrf2) is a key transcription factor in cellular antioxidant responses and a major ferroptosis signaling molecule in the nucleus [106, 107]. Under oxidative stress, Nrf2 dissociates from its repressor Keap1, translocates to the nucleus, and activates a battery of cytoprotective genes [108]. Nrf2 comprehensively controls ferroptosis by transcriptionally regulating multiple components of the antioxidant network, including glutathione synthetase, SLC7A11, and GPX4 [109]. This positions Nrf2 as a crucial upstream regulator of the System Xc-/GSH/GPX4 axis in AS. Experimental evidence confirms that activating Nrf2 and promoting its nuclear translocation enhances the SLC7A11/GPX4 signaling pathway, inhibits lipid peroxidation, and alleviates vascular endothelial ferroptosis [110]. Furthermore, Nrf2’s regulation extends beyond GPX4. Recent studies identify the CoQ-FSP1 axis as a key downstream effector of Nrf2 [111], and Nrf2 also regulates iron metabolism by controlling ferritin synthesis and degradation (ferritinophagy) [112, 113]. Nrf2 deficiency leads to dysregulated iron storage and increased sensitivity to ferroptosis [114]. Due to its dual roles in regulating antioxidant defense and iron metabolism, Nrf2 is a key node in AS-related ferroptosis.

However, the role of Nrf2 in AS remains controversial and context-dependent. While its antioxidant functions are generally considered atheroprotective, sustained or excessive Nrf2 activation has been linked to pro-atherogenic effects in certain models. This duality is evident in several key findings. For instance, Nrf2 can directly upregulate the scavenger receptor cluster of differentiation 36 (CD36) *in vitro*, promoting oxLDL uptake in macrophages and foam cell formation. Additionally, Nrf2 in bone marrow-derived cells is involved in late-stage atherosclerotic plaque formation, potentially influencing inflammatory responses within plaques [115].

Conversely, opposing evidence highlights a protective role. Specific knockout of *Nrf2* in macrophages of LDLR^-⁣/-^ mice exacerbated lesions, accompanied by enhanced inflammation and foam cell formation [116]. Moreover, Nrf2 has been reported to influence the proliferation and migration of VSMCs, which could benefit plaque stability in certain contexts [117]. This suggests that the ultimate effect of Nrf2 in AS may depend on the cell type and pathological microenvironment in which it acts.

## 4. GPX4: A Promising Therapeutic Target for AS

Given the established role of ferroptosis in promoting AS, inhibiting it offers a viable therapeutic approach. In this context, GPX4 emerges as a highly promising anti-ferroptotic target for AS intervention. Experimental evidence strongly supports this potential: Guo *et al*. [14] demonstrated that GPX4 overexpression in an AS mouse model inhibits oxidized lipid deposition, reduces vascular cell reactivity, and impedes monocyte adhesion by suppressing vascular cell adhesion molecule-1. Clinically, GPX4 expression is notably decreased in advanced human atherosclerotic plaques [118], and endothelial-specific *GPX4* knockout leads to endothelial dysfunction and increased thrombosis risk [119]. *In vitro* studies further reveal that pro-atherogenic stimuli like ox-LDL can induce GPX4 suppression and subsequent ferroptosis in macrophages [120]. Beyond its core role in curbing lipid peroxidation, GPX4 also modulates inflammatory pathways by inhibiting LOX and COX activity, thereby attenuating plaque development [121, 122].

### 4.1 Interplay Among GPX4, Ferroptosis, and Immune-Inflammation

To fully appreciate GPX4’s therapeutic potential in AS, it is essential to understand its function within the broader context of chronic inflammation, a hallmark of the disease. The imbalance between oxidation and anti-oxidation can disrupt cell signaling, and oxidative stress closely interacts with innate immune signaling pathways [123]. Pro-inflammatory signaling, potentially initiated via toll/interleukin-1 receptor (TIR) domains [124], generates ROS that suppresses GPX4 function and promotes ferroptosis. In turn, DAMPs released from ferroptotic cells further activate immune cells, sustaining a pro-inflammatory microenvironment that exacerbates plaque development and instability [125]. This creates a pathogenic cycle central to AS progression. Thus, targeting GPX4 not only regulates redox balance but may also indirectly modulate the immune microenvironment within plaques, addressing both oxidative cell death and chronic inflammation, which are closely linked drivers of AS.

### 4.2 Diverse Modulators of the GPX4 Pathway

The therapeutic landscape targeting the GPX4 pathway is rapidly expanding, moving beyond classical ferroptosis inducers (e.g., RSL3, ML162) that suffer from low specificity and poor pharmacokinetics [126, 127]. As summarized in Table 1 (Ref. [9, 14, 26, 62, 110, 112, 128, 129, 130, 131, 132, 133, 134, 135]), numerous innovative compounds and strategies have demonstrated efficacy in AS models by directly or indirectly enhancing GPX4 function.

**Table 1.** **Compounds/strategies modulating GPX4 and related pathways and their potential roles in atherosclerosis**.

Compound/Intervention strategy	Mechanism of action	Effect on GPX4	Biological effects in AS model	Reference
*GPX4* gene overexpression	Inhibits lipid peroxidation	↑	Significantly reduced plaque area in aortic tree and sinus; decreased vascular cell sensitivity to oxidized lipids	Guo *et al*. 2008, [14]
High uric acid	Inhibits Nrf2 and autophagy	↓	Promoted foam cell formation; exacerbated plaque progression, mitochondrial damage, and ferroptosis	Yu *et al*. 2022, [112]
Micheliolide	Targets KEAP1/Nrf2 interaction; enhances Nrf2 nuclear translocation	↑	Significantly slowed AS plaque progression; improved mitochondrial function and ferroptosis; reduced inflammation and ROS	Luo *et al*. 2023, [9]
Ferritin heavy chain	Regulates iron metabolism	↑	Improved blood glucose and lipid levels; improved aortic structure and iron deposition; inhibited ROS and inflammation	Yuan *et al*. 2023, [26]
si-PVT1	Regulates microRNA-106b-5p/ACSL4 axis	↑	Reduced AS plaque area and lipid deposition; improved ferroptosis markers	Zhang *et al*. 2023, [133]
Hydroxysafflor Yellow A	Regulates miR-429/SLC7A11 pathway	↑	Effectively reduced AS plaque formation in T2DM/AS mice; inhibited HUVEC ferroptosis	Rong *et al*. 2023, [128]
*GPX4* gene overexpression	Inhibits lipid peroxidation	↑	Reduced lipid peroxidation in ApoE⁻/⁻ mouse plaques, but did not alter plaque size or composition	Coornaert *et al*. 2024, [132]
Astaxanthin-loaded polylactic acid-glycolic acid nanoparticles	Activates Nrf2/SLC7A11/GPX4 pathway	↑	Significantly improved AS progression and markers of ferroptosis and oxidative stress	Jin *et al*. 2025, [130]
Ferrostatin-1	Activates AMPK pathway regulating iron and lipid metabolism	↑	Alleviated aortic AS lesions and foam cell formation; reduced macrophage lipid accumulation and inflammation	Yang *et al*. 2025, [62]
Polydopamine nanoparticles	Scavenges ROS; regulates iron/lipid metabolism; upregulates GPX4/ Nrf2 pathway	↑	Reduced plaque area; enhanced fibrous cap stability; reduced macrophage infiltration; downregulated inflammation; inhibited ferroptosis	Dai *et al*. 2025, [131]
Dapagliflozin	Activates RAP1B/Nrf2/GPX4 pathway; promotes mitochondrial biogenesis	↑	Improved mitochondrial function; reduced plaque area and endothelial ferroptosis	Zhu *et al*. 2025, [110]
CD16/32-ZIF90@Sp	Targets macrophages via CD16/32; ZIF90 releases Sp improving mitochondrial function	↑	Effectively slowed AS progression and intra-plaque macrophage ferroptosis in ApoE⁻/⁻ mice	Chen *et al*. 2025, [134]
Capsiate	Activates Nrf2/GPX4/SLC7A11 pathway; modulates gut microbiota	↑	Reduced aortic plaque area and lipid levels; increased gut microbiota diversity and beneficial bacteria abundance	Shen *et al*. 2025, [129]
Baicalin	Improves gut microbiota and brain lipid metabolism	↑	Improved depressive-like behavior in mice; reduced aortic plaque area	Ren *et al*. 2025, [135]

AS, atherosclerosis; KEAP1, kelch-like ECH-associated protein 1; ACSL4, acyl-CoA synthetase long-chain family member 4; SLC7A11, solute carrier family 7 member 11; AMPK, AMP-activated protein kinase; RAP1B, Ras-related protein 1B; CD16/32, cluster of differentiation 16/32; ZIF90, zeolitic imidazolate framework-90.

Many phytochemicals (e.g., Baicalin [97], Hydroxysafflor Yellow A [128], Capsiate [129]), and existing drugs (e.g., Dapagliflozin [110]) converge on the Nrf2/GPX4 axis, enhancing the endogenous antioxidant response to confer protection. Strategies such as astaxanthin-loaded polylactic acid-glycolic acid nanoparticles [130] and polydopamine nanoparticles [131] therapy utilize nanoparticles for targeted delivery and improved bioavailability, overcoming the limitations of systemic administration. Gene and RNA targeting strategies, including *GPX4* gene overexpression [132] and si-PVT1 [133], demonstrate the potential of directly manipulating expression networks to suppress ferroptosis, effectively upregulating GPX4 and improving AS.

However, the available evidence reveals complexities and apparent contradictions. Notably, while Guo *et al*. [14] found that GPX4 overexpression significantly reduced plaque area, a more recent study by Coornaert *et al*. [132] reported that it reduced lipid peroxidation without altering plaque size or composition. The timing of intervention, specific cell types targeted, and the model systems used may account for these divergent outcomes, suggesting that the therapeutic window and functional outcomes of GPX4 modulation are context-dependent. The future gene therapy approaches must be carefully tailored to the pathological context.

A summary of the mechanisms reveals that current strategies, whether involving natural small molecules or repurposed drugs, predominantly modulate GPX4 indirectly via its upstream regulators like Nrf2 and SLC7A11. There is a notable scarcity of research directly targeting the GPX4 protein itself. Most studies focus merely on the outcome of increased protein expression, with limited investigation into the processes of its transcription, translation, and activation. Furthermore, the field is heavily biased towards Nrf2-dependent mechanisms. While activating this master regulator is effective, its broad effects on numerous genes raise concerns about potential off-target consequences and a lack of pathway specificity. The repeated validation of this axis, though supportive, also indicates a lack of mechanistic diversity in current approaches.

Finally, systemic enhancement of a powerful antioxidant defense could potentially disrupt redox-dependent physiological signaling. For cardiovascular disease (CVD) treatment, which often requires long-term medication, this raises serious concerns about potential interference with normal immune cell function and other redox-sensitive processes, underscoring the critical need for targeted approaches. To avoid systemic side effects, particularly crucial for chronic CVD treatments, there is a pressing need for cell- and site-specific targeting to confine the therapeutic action to the atherosclerotic plaque microenvironment.

### 4.3 Limitations

Despite compelling preclinical data, translating GPX4-targeted therapies into clinical practice faces significant hurdles that must be proactively addressed in future research.

The historical lack of direct GPX4 agonists, primarily due to the absence of a well-defined conventional binding pocket, remains a key obstacle in the field. Although the discovery of an allosteric site offers new insights [136], no *in vivo* studies specifically investigating GPX4 activation in AS models have been reported to date. This limitation directly impacts the translational potential of GPX4-based therapies for cardiovascular disease.

Selenium supplementation offers another potential avenue to enhance the activity of selenoproteins, including GPX4. Increasing selenium levels can improve endothelial dysfunction and suppress AS lesions [137]. However, we critically note that most studies report effects on general GPx enzyme activity rather than specifically on GPX4, leaving the precise contribution of GPX4 to selenium-mediated cardiovascular protection unclear.

Current research on GPX4 regulation in AS is heavily reliant on indirect pathways, predominantly through upstream regulators such as Nrf2. While viable, this approach lacks specificity and may lead to off-target effects due to the broad regulatory network of these upstream molecules. Additionally, the majority of existing studies have focused on single-pathway interventions, with limited exploration of potential synergistic effects from combination strategies. This narrow focus may overlook more effective therapeutic approaches that target multiple ferroptosis-related pathways simultaneously.

## 5. Conclusions

In conclusion, GPX4 represents a cornerstone target in combating ferroptosis-driven atherosclerosis, with this pathway demonstrating significant translational viability. The growing arsenal of strategies—including precision-targeted nanoparticle delivery systems, GPX4 mimetics such as Compd.5 [138], and selenium supplementation—offers promising avenues to overcome current limitations and translate GPX4-based interventions toward clinical application. Future research should prioritize the development of cell-specific delivery systems to achieve plaque-targeted therapy, the exploration of combination strategies that simultaneously target multiple ferroptosis-related pathways, and rigorous *in vivo* validation in advanced AS models. By addressing these challenges, GPX4-based therapies hold substantial promise for improving the management of atherosclerotic cardiovascular disease.
